# RGS14 promotes the progression of hepatocellular carcinoma by activating the cAMP/PKA/CREB signaling pathway

**DOI:** 10.1007/s00432-025-06212-y

**Published:** 2025-05-02

**Authors:** Xiangnan Liang, Bin Xu, Qiuxiang Wang, Kai Gong, Chun Han, Binwen Sun, Kexin Ma, Liming Wang

**Affiliations:** 1 Engineering Research Center for New Materials and Precision Treatment Technology of Malignant Tumors Therapy, The Second Affiliated Hospital of Dalian Medical University, Dalian, 116023 China; 2 Engineering Technology Research Center for Translational Medicine, The Second Affiliated Hospital of Dalian Medical University, Dalian, 116023 China; 3 Division of Hepatobiliary and Pancreatic Surgery, Department of General Surgery, The Second Affiliated Hospital of Dalian Medical University, 467 Zhongshan Road, Dalian, Liaoning 116023 China

**Keywords:** RGS14, HCC, cAMP/PKA/CREB signaling pathway, Prognosis, Biomarker

## Abstract

**Background:**

G protein-coupled receptors (GPCRs) mediate the intracellular signals that drive tumor development. Regulator of G protein signaling 14 (RGS14), a key negative regulator of GPCR signaling, influences liver injury, fat metabolism, and inflammation. However, the role of RGS14 in hepatocellular carcinoma (HCC) progression and its underlying mechanisms remain unclear.

**Methods:**

In this study, we compared three pairs of HCC tissues and matched portal vein tumor thrombus (PVTT) samples using 4D-FastDIA proteomics to identify differentially expressed proteins. The clinical significance of RGS14 expression was further evaluated in HCC patient cohorts. Stable RGS14-overexpressing/knockdown cell models were established for functional assays (CCK-8, colony formation, Transwell, and wound healing assays). Additionally, tumor proliferation was evaluated through in vivo studies using a subcutaneous xenograft mouse model. RNA sequencing and western blot analysis were subsequently applied to validate the potential downstream signaling pathways.

**Results:**

The results revealed that RGS14 was overexpressed in HCC tissues, which was correlated with adverse clinical outcomes. We also confirmed that RGS14 increased the proliferation, colony formation, migration, and invasion and promoted the epithelial‒mesenchymal transition (EMT) of HCC cells both in vitro and in vivo. Mechanistically, RGS14 elevated intracellular cAMP levels, activating the PKA/CREB axis to drive HCC progression.

**Conclusion:**

Our findings suggest that RGS14 plays a critical oncogenic role in HCC by regulating cAMP/PKA/CREB pathway activation, underscoring its potential as both a prognostic marker and therapeutic target for HCC patients.

**Supplementary Information:**

The online version contains supplementary material available at 10.1007/s00432-025-06212-y.

## Introduction

Primary liver cancer (PLC) imposes a significant global health burden (Yang et al. 2019), ranking as the sixth most common cancer and the third leading cause of cancer-related mortality worldwide (Bray et al. 2024). Hepatocellular carcinoma (HCC), which accounts for 75–80% of PLC cases, is predominantly associated with viral hepatitis (Rumgay et al. 2022). Despite advances in HCC treatment, challenges such as late diagnosis, chemotherapy resistance, frequent recurrence, and intrahepatic/extrahepatic metastases remain major contributors to its poor prognosis (Oura et al. 2021; Wang et al. 2019). The molecular mechanisms driving HCC progression and metastasis are incompletely understood, hampering the development of effective therapies. Consequently, identifying novel mechanisms and therapeutic targets within the complex regulatory network of HCC is critically needed.

Regulators of G protein signaling (RGSs) constitute a critical protein family that regulates G protein-coupled receptor (GPCR) signal transduction (Watson et al. 1996). These proteins bind to the α subunit of heterotrimeric G proteins, accelerating GTP-to-GDP hydrolysis to terminate downstream signaling cascades (Hollinger and Hepler 2002). Therefore, RGS proteins are considered important downstream nodes in GPCR signaling (Masuho et al. 2020). Dysregulated RGS expression is implicated in various pathologies. RGS17 may contribute to cisplatin-induced ototoxicity through immune cascade activation, highlighting its potential as a therapeutic target (Al Aameri et al. 2025). Accumulating evidence suggests that RGS genes may act as tumor suppressor genes or oncogenes in a variety of cancers (Li et al. 2023). For example, RGS10 is recognized as a tumor suppressor that inhibits the growth and metastasis of breast cancer cells (Liu et al. 2024). RGS10 suppression promotes colorectal cancer progression, while DNA methylation inhibition may enhance COAD patient survival (Caldiran and Cacan 2022). In contrast, in bladder cancer, RGS20 upregulation induces cancer cell proliferation and migration (Li et al. 2019). Notably, the malignant properties of RGSs in HCC and the underlying mechanisms involved remain poorly characterized.

Regulator of G protein signaling 14 (RGS14), a member of the RGS D/R12 family, contains a common 130-amino acid RGS domain, a GoLoco/GPR (GL) motif and a tandem (R1 and R2) Ras/Rap binding domain (RBD) (Shu et al. 2010). The GL motif binds to an inactive form of Giα1/3 (designated GNAI1/3) to form a stable complex that exhibits GDP dissociation inhibitor (GDI) activity (Coleman et al. 1994). Previous studies have focused mostly on the regulation of RGS14 in postsynaptic signal transduction in brain and spinal cord plasticity (Harbin et al. 2021; Vellano et al. 2011), as well as in benign liver diseases such as hepatic ischemia‒reperfusion injury (Zhang et al. 2022). However, the expression and function of RGS14 in HCC remain largely unknown.

In this study, we observed that RGS14 expression was markedly elevated in HCC, and its upregulation correlated with unfavorable clinical outcomes in patients. Functional experiments further revealed that RGS14 promotes HCC cell proliferation and metastatic potential. Mechanistically, we found that RGS14 exerts its oncogenic effects by regulating cyclic adenosine monophosphate (cAMP) levels and activating the downstream protein kinase A (PKA)/cAMP response element-binding protein (CREB) signaling pathway. These findings support RGS14 as both a potential prognostic biomarker and a promising therapeutic target for HCC.

## Materials and methods

### Patients and tissue samples

A total of 88 paired tumor and adjacent nontumorous tissue samples were collected from HCC patients who underwent curative hepatectomy. Pathologists confirmed that the surgical samples were HCC samples. The diagnosis of liver cirrhosis was classified according to the METAVIR scoring system (Bedossa and Poynard 1996), which evaluates the presence or absence of pseudolobular formation. Prior to surgery, no patients had received nonsurgical therapies. These samples were subsequently utilized to construct tissue microarrays (TMAs). Three pairs of portal vein tumor thrombus (PVTT) and HCC samples were subjected to 4D-FastDIA proteomics to identify differentially expressed proteins. The clinicopathologic characteristics of these samples are summarized in Table 1. The use of all tissue samples was approved by the Ethics Committee of the Second Affiliated Hospital of Dalian Medical University (Study identifier 2022 − 166).

**Table 1:** Correlation between the clinicopathologic characteristics and RGS14 expression in HCC

Characteristics		*N* = 88	RGS14 expression		P valule
			Low (*N = 45*)	High (*N = 43*)	
Sex (male)	Male	69	32	37	0.089
	Female	19	13	6	
Age (year)	>60	40	19	21	0.553
	≤ 60	48	26	22	
AFP level (ng/ml)	≥ 20	42	22	20	0.823
	<20	46	23	23	
Tumor size (cm)	≥ 5	26	14	12	0.742
	<5	62	31	31	
Tumor number	1	58	31	27	0.546
	≥ 2	30	14	16	
Cirrhosis	Absent	24	13	11	0.728
	Present	64	32	32	
HBV infection	Absent	20	11	9	0.694
	Present	68	34	34	
Venous infiltration	Absent	36	21	15	0.261
	Present	52	24	28	
TNM stage	I + II	73	41	32	0.037*
	III + IV	15	4	11	
Satellite nodule	Absent	68	34	34	0.694
	Present	20	11	9	
Degree of differentiation	Low	28	16	12	0.441
	high-moderate	60	29	31	

*Abbreviations*: AFP, alpha-fetoprotein; HBV, hepatitis B virus; TNM, tumor-node-metastasis

*Indicates statistically significant

### Analysis of publicly available data

The expression patterns of RGS14 in HCC tissues and the corresponding clinical information were obtained from the Cancer Genome Atlas (TCGA) online database (http://portal.gdc.com). R software V4.0.3 was used for statistical analysis.

### Cell culture and transfection

The SNU387, SNU449, MHCC97H, Huh7, Hep3B, and HepG2 cell lines were purchased from the Cell Bank of the Chinese Academy of Sciences (Shanghai, China), and the cell lines were identified by STR profiling. SNU387 and SNU449 cells were cultured in RPMI 1640 medium supplemented with 10% fetal bovine serum (FBS) (Gibco, USA), and MHCC97H and Huh7 cells were cultured in high-glucose DMEM supplemented with 10% FBS. Hep3B and HepG2 cells were cultured in MEM supplemented with 10% FBS. All the cell lines were cultured in an atmosphere of 5% carbon dioxide at 37 °C. RGS14-targeting shRNA (shRNA1, shRNA2, and shRNA3), negative control (LV3-NC) shRNA, RGS14-overexpressing lentivirus and negative control lentivirus (LV5-NC) were obtained from Gemma Corporation (Shanghai, China). HCC cells were transfected separately according to the manufacturer’s protocol, the transfected cells were then screened with puromycin (2 µg/ml) for 2 weeks, and the efficiency of overexpression or knockdown was assessed by western blotting. The sequences of the shRNAs are presented in Table S1.

### Cell viability and colony formation assays

Cell viability was determined with a Cell Counting Kit-8 (CCK-8) (MCE, USA). In brief, cells were seeded at a density of 2 × 10^3^ cells per well in 96-well plates containing 100 µL complete medium, and then, the cells were cultured for 24 h. Ten microliters of CCK-8 reagent was added to each well daily, and the cells were incubated for 2 h. The optical density at 450 nm was measured with a microplate spectrophotometer (Bio-Rad). The experiment was performed in duplicate three independent times.

For the colony formation assay, the cells were evenly spread in 6-well plates (1 × 10^3^ cells per well). The cultures were incubated for 2 weeks, and when visible colonies had formed, the cells were fixed with 4% paraformaldehyde for 20 min, stained with 1% crystal violet for 10 min, photographed and counted.

### Migration and cell invasion assays

For the cell migration experiment, 2.5 × 10^4^ cells were seeded in the upper chamber of a Transwell plate (Corning, USA), and 650 µL of culture medium supplemented with 20% FBS was added to the lower chamber as a chemotactic agent. To assess cell invasion, Matrigel (BD, USA) was added to the Transwell chamber before the cells were seeded. The cells were incubated at 37 °C for 24 or 48 h, the cells in the upper chamber were removed, and then, the cells that had migrated or invaded were fixed with 4% paraformaldehyde. The fixed cells were stained with crystal violet. The data were analyzed using ImageJ software (NIH, USA).

### Protein extraction and western blotting

Total proteins were isolated from cells and tissues with RIPA lysis buffer (Beyotime) supplemented with a protease inhibitor solution (Beyotime) and a phosphatase inhibitor solution (TargetMol). A bicinchoninic acid (BCA) assay kit (Thermo, USA) was used to determine the protein concentrations of the lysates. Denatured proteins (30 µg of protein/lane) in the lysate supernatants were separated on SDS‒polyacrylamide gels and then transferred to polyvinylidene fluoride (PVDF) membranes (Millipore, USA). After being blocked in rapid blocking solution (NCM, China) at room temperature for 15 min, the PVDF membranes were incubated with primary antibodies overnight at 4 °C. The PVDF membranes were then incubated with the secondary antibody for 1 h at 37 °C, and the protein bands were visualized with a hypersensitive chemiluminescence reagent. Image software was used to quantify protein levels from three independent experiments. Details about the antibodies used are provided in Table S2.

### cAMP measurements

Adherent cells were washed with precooled PBS and harvested after trypsin digestion. PBS supplemented with protease inhibitors (100 µl to 200 µl for every 1 × 10^6^ cells) was added, the cells were lysed with an ultrasonic homogenizer, and the supernatants were collected for analysis. The intracellular cAMP levels were determined by measuring the absorbance at 450 nm with a cAMP ELISA kit (Elabscience, China) according to the manufacturer’s instructions and by comparing the absorbance of each sample to a standard curve.

### Immunohistochemistry (IHC) and scoring

Protein expression in 88 human HCC tissues was measured by IHC according to a previously described experimental protocol (Jiang et al. 2020). The staining results were scored and evaluated separately by two experienced pathologists in a blinded manner, and the proportion of positive cells as well as the staining intensity were evaluated to obtain the final score. The proportion of positively stained cells (negative, score of 0; 1–25%, score of 1; 25–50%, score of 2; 50–75%, score of 3; and 75–100%, score of 4) and staining intensity (no staining, score of 0; weak staining, score of 1; moderate staining, score of 2; strong staining, score of 3) were used to score positive staining. Finally, the staining index (SI) was calculated as follows: SI = staining intensity score × positive staining proportion score. Sections with a total score < 4 were considered the low RGS14 expression group, and sections with a total score ≥ 4 were considered the high RGS14 expression group.

### RNA sequencing

MHCC97H cells were divided into shNC and shRGS14 groups. The cells from each group were harvested and sent to Servicebio (Wuhan, China) for transcriptome sequencing. Detailed information regarding the sequencing process can be obtained by contacting the company’s customer support department.

### Tumor xenograft model

The animal experiments were approved by the Institutional Animal Care and Use Committee of Dalian Medical University (2024236). Four-week-old female BALB/c nude mice were housed in a specific pathogen-free (SPF) animal facility. Ten mice were randomly assigned to two groups. Each mouse received a subcutaneous injection of 5 × 10^6^ MHCC97H cells, either with or without RGS14 knockdown, into the right flank (200µL), to establish a hepatocellular carcinoma (HCC) xenograft model. The body weights and tumor sizes of the mice were monitored weekly, and their volumes were calculated using the following formula: V (mm³) = 1/2 × length × width². After two weeks, the mice were euthanized, and the tumors were excised, weighed, and subsequently paraffin-embedded for IHC analysis.

### Statistical analysis

SPSS 26.0 (SPSS, Chicago, USA) software was used for statistical analysis. Statistical differences were analyzed by two-tailed Student’s t test or analysis of variance, where applicable. Correlations between RGS14 expression levels and clinicopathological parameters were evaluated via the chi-square test and Fisher’s exact test. Survival curves were generated using the Kaplan‒Meier method and analyzed using the log-rank test. Univariate and multivariate analyses were performed with the Cox proportional hazards regression model to determine the prognostic factors. A P value of less than 0.05 was considered to indicate a statistically significant difference.

## Results

### RGS14 is highly expressed in HCC

**Fig. 1:**
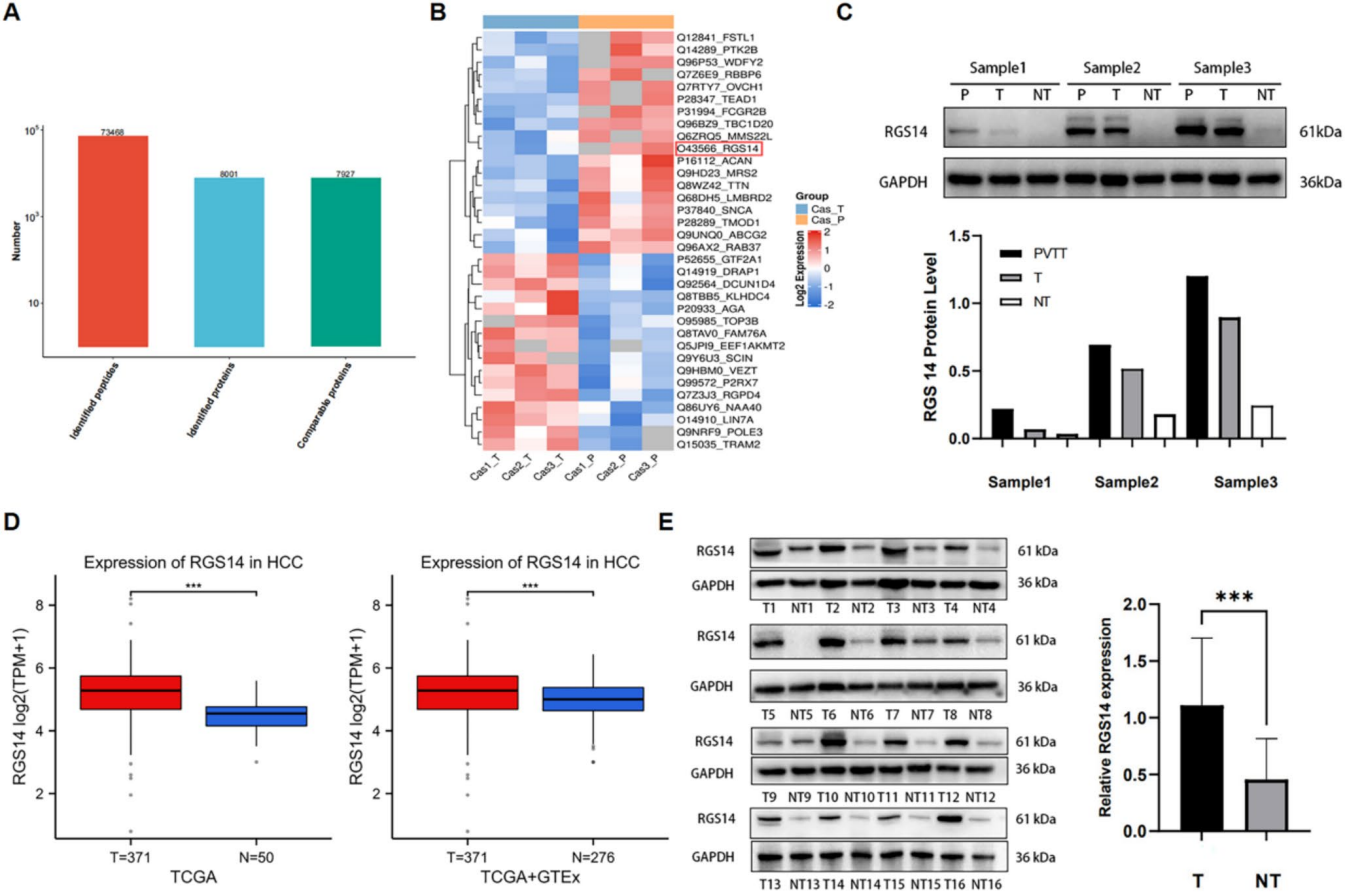
Expression of RGS14 in Adjacent Non-Tumor Tissues, HCC, and PVTT. (**A**) Number of proteins identified by 4D-FastDIA proteomics. (**B**) Heatmap illustrating the top upregulated and downregulated proteins in PVTT compared with matched HCC tissues. (**C**) Western blotting analysis of RGS14 expression in PVTT, HCC, and adjacent non-tumor tissues. (**D**) Differential expression analysis of RGS14 mRNA between HCC tumors and adjacent normal tissues based on TCGA and GTEx databases. (**E**) Western blotting analysis of RGS14 expression in 16 pairs of HCC and matched adjacent non-tumor tissues. Statistical significance was assessed using a two-tailed unpaired Student’s t-test. *** *P* < 0.001

PVTT, a prevalent complication of HCC, promotes tumor progression by facilitating metastatic dissemination and conferring adverse clinical outcomes (Zhang et al. 2023). We used 4D-FastDIA proteomics technology to analyze 3 pairs of HCC tissue samples and matched PVTT tissue samples to identify differentially expressed proteins. After data retrieval and filtering, 73,468 peptides and 7,927 comparable proteins were ultimately identified (Fig. 1A). According to the screening criteria (fold change > 1.5 and *P* < 0.05), 34 differentially expressed proteins were identified (Table S3). Subsequent functional categorization and statistical evaluation of the differentially expressed proteins between the two groups (Supplementary Fig. 1) established their predominant regulatory roles in biological process modulation, signal transduction cascades, and transcriptional control mechanisms. Among the top 10 upregulated proteins (Fig. 1B), RGS14, a critical modulator of GPCR-Gα signaling cascades, has pivotal functions in promoting tumor progression. Western blotting analysis revealed that RGS14 expression gradually increased in tumor-adjacent tissues, HCC tissues, and PVTT tissues (Fig. 1C). Analysis of publicly accessible databases revealed markedly elevated RGS14 mRNA levels in tumor samples compared with those in control samples across both the TCGA and GTEx datasets (Fig. 1D). Similar to RGS14 mRNA expression, RGS14 protein expression was significantly higher in 16 pairs of HCC tissues than in tumor-adjacent tissues, as shown by immunoblotting (Fig. 1E). These results indicate that RGS14 expression is significantly increased in HCC at both the mRNA and protein levels.

### Increased RGS14 expression is associated with clinical features and predicts a poor prognosis in HCC patients

**Fig. 2:**
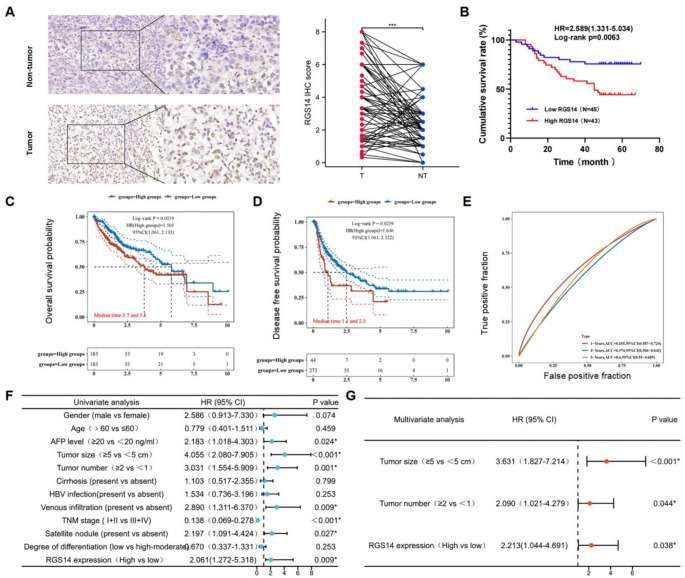
Expression and Clinical Significance of RGS14 in Hepatocellular Carcinoma Tissues. (**A**) Representative immunohistochemical images (left panel) and statistical analysis (right panel) of RGS14 expression in a liver cancer tissue microarray (*N* = 88). Scale bar, 100 μm. (**B**) Kaplan–Meier curves illustrated the overall survival of 88 HCC patients categorized by high versus low RGS14 expression levels (log-rank test, *p* = 0.0063). (**C**, **D**) TCGA-LIHC data showed that high RGS14 expression was associated with reduced OS and DFS. (**E**) The ROC curves and AUC values of RGS14 at different time points. (**F**, **G**) Cox regression analyses evaluated the correlation between RGS14 expression and prognosis. *** *P* < 0.001

Next, to determine whether RGS14 expression was associated with HCC progression, immunohistochemical staining was performed on 88 pairs of HCC tissue and nontumor tissue microchips (representative image in Fig. 2A) to analyze the correlation between RGS14 expression and the clinical parameters of HCC. We analyzed the relationships between RGS14 expression and the clinical parameters of HCC patients according to the tissue microarray IHC scores. As shown in Table 1, RGS14 expression was significantly correlated with the TNM stage. Survival analysis demonstrated that patients with high RGS14 expression presented significantly reduced overall survival (OS) (Fig. 2B). Analysis of the TCGA cohort revealed that elevated RGS14 expression was correlated with poorer OS and disease-free survival (DFS) (Fig. 2C, D). ROC curve analysis confirmed the prognostic value of RGS14, with AUC values of 0.655, 0.574, and 0.6 for 1-year, 3-year, and 5-year survival prediction, respectively (Fig. 2E). Univariate analysis revealed that the AFP level, tumor size, tumor number, TNM stage, vascular invasion, satellite lesions, and RGS14 expression were significantly associated with OS in HCC patients (Fig. 2F). In addition, multivariate analysis revealed that only the RGS14 level, tumor number, and tumor size were independent predictors of OS in HCC patients (Fig. 2G). These results indicate that high RGS14 expression is significantly associated with poor prognosis in HCC patients, and that as a risk factor that affects HCC metastasis, high RGS14 expression may play an important role in HCC malignant progression.

### RGS14 promotes HCC cell growth in vitro

**Fig. 3:**
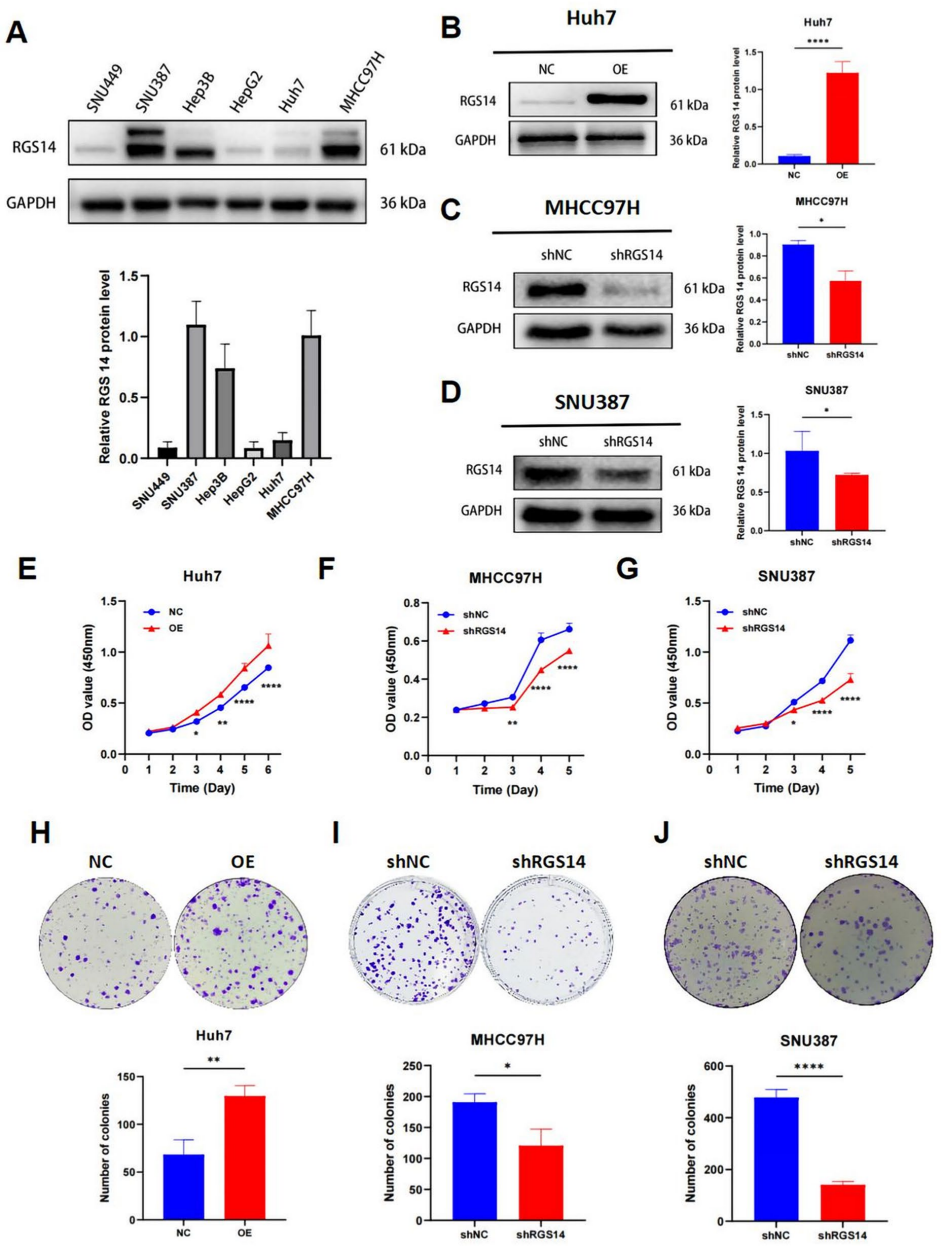
RGS14 promotes the proliferation of HCC cells in vitro. (**A**) The expression levels of RGS14 in different HCC cells were detected by Western blot. (**B**-**D**) Validation of overexpression and knockdown of RGS14 protein levels via Western Blot analysis. (**E**-**G**) CCK-8 assay to evaluate the impact of RGS14 knockdown and overexpression on HCC cell proliferation. (**H**-**J**) Colony formation assay to assess cell proliferation in HCC Cells. Representative images of colonies are shown in the upper panel, and the number of colonies was quantified in the lower panel. Data are shown as mean ± SD from three independent experiments. Statistical significance was determined using a two-tailed unpaired t-test. * *P* < 0.05, ** *P* < 0.01, *** *P* < 0.001, **** *P* < 0.0001

To investigate the biological function of RGS14 in HCC cells, based on RGS14 expression in HCC cell lines (Fig. 3A), we used lentiviral technology to establish stable models of RGS14 overexpression in the Huh7 cell line and RGS14 knockdown in the MHCC97H and SNU387 cell lines (Fig. 3B-D). In cultured Huh7 cells, RGS14 overexpression increased cell viability, whereas RGS14 knockdown had the opposite effect (Fig. 3E-G). In addition, RGS14 overexpression promoted colony formation, whereas RGS14 knockdown inhibited colony formation (Fig. 3H-J).

### RGS14 promotes migration and invasion in HCC

**Fig. 4:**
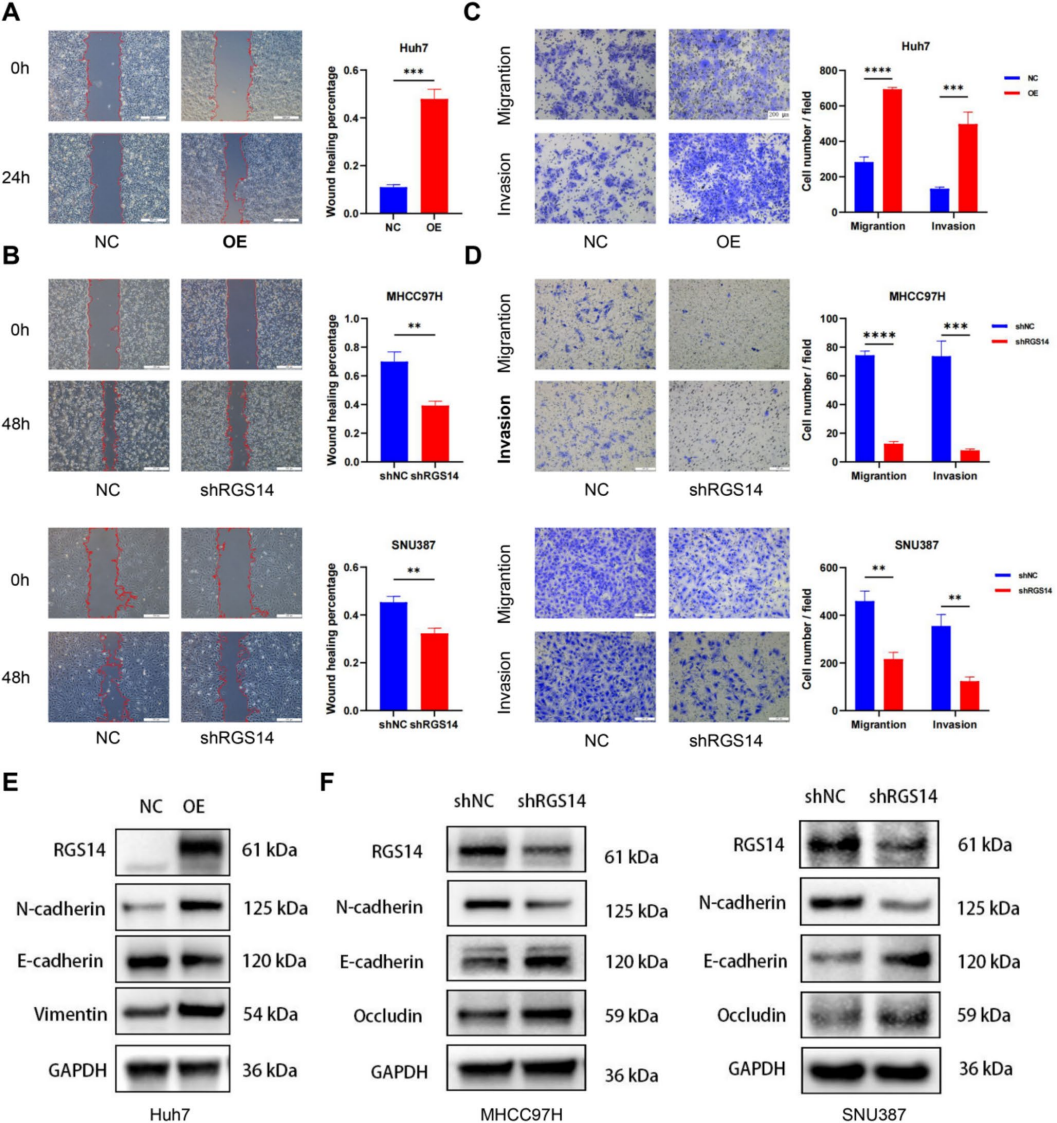
Effect of RGS14 on HCC Cell Migration and Invasion. (**A**, **B**) Wound healing assay to evaluate the effect of RGS14 expression on the migration of HCC cells. Scale bar, 200 μm. (**C**, **D**) The effects of RGS14 knockdown and overexpression on the migration and invasion abilities of HCC cells were evaluated by Transwell assay. Representative images (left panel) and summary bar graphs (right panel) are shown. Scale bar, 200 μm. (**E**, **F**) Western blotting analysis of the expression of EMT-related proteins in HCC cells. Data are shown as mean ± SD from three independent experiments. ** *P* < 0.01, *** *P* < 0.001, **** *P* < 0.0001

To determine the effect of RGS14 on cell migration ability, we performed a wound-healing assay, which revealed that RGS14 overexpression increased HCC cell migration, whereas RGS14 knockdown inhibited cell migration (Fig. 4A, B). Transwell assays also revealed that RGS14 increased cell invasion and migration potential (Fig. 4C, D). Considering that EMT is an obvious mechanism that increases HCC cell progression and metastasis, we examined the expression of several EMT markers (Alqurashi et al. 2023). As shown by western blotting, RGS14 knockdown inhibited the expression of mesenchymal markers (N-cadherin) and upregulated the expression of epithelial markers (E-cadherin and Occludin) in SNU387 and MHCC97H cells. In contrast, in Huh7 cells, the overexpression of RGS14 downregulated the expression of E-cadherin while increasing the expression of N-cadherin and Vimentin (Fig. 4E, F; Supplementary Fig. 2A-C). In conclusion, RGS14 plays a critical role in promoting the migration, invasion, and EMT of HCC cells.

### RGS14 upregulation activates the cAMP/PKA/CREB signaling pathway in HCC

**Fig. 5:**
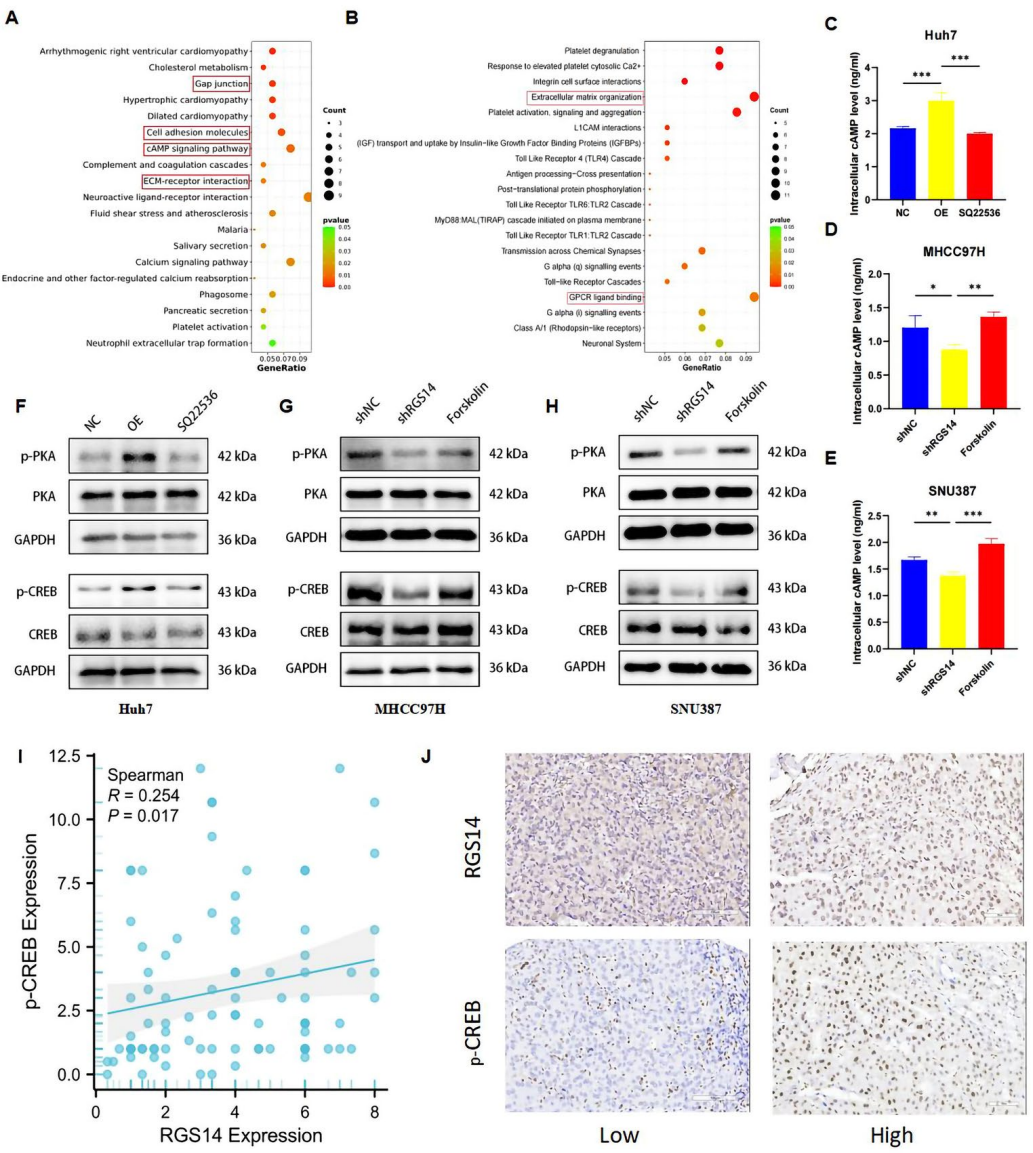
RGS14 activates the cyclic adenosine monophosphate (cAMP) signaling pathway in HCC. (**A**) KEGG pathway enrichment analysis of differentially expressed genes. (**B**) Reactome pathway enrichment analysis of differentially expressed genes. (**C**) Intracellular cAMP levels in Huh7 cells (RGS14 control, overexpression, and SQ22536-treated overexpression groups with 10µmol/L adenylate cyclase inhibitor) were evaluated by ELISA. (**D**, **E**) Changes in intracellular cAMP levels were assessed in three HCC cell groups: negative control, shRGS14, and shRGS14 treated with 10µmol/L adenylate cyclase agonist Forskolin. (**F**) Western blotting was performed to examine the expression of cAMP pathway-related proteins in three Huh7 cell groups: RGS14 control, overexpression, and SQ22536-treated overexpression (10µmol/L). (**G**, **H**) The alterations in cAMP pathway-related protein expression were evaluated by Western blotting across three HCC cell subgroups: negative control, shRGS14, and shRGS14 exposed to 10µmol/L Forskolin. (**I**) Scatterplots with fitting line show a positive correlation between RGS14 and p-CREB expression in HCC tissues. Spearman correlation analysis provides the correlation coefficient (*R*) and P value. (**J**) Representative immunohistochemical staining images of HCC tissues in the RGS14 and p-CREB groups (Scale bar, 100 μm). Data are shown as mean ± SD from three independent experiments. Data were subjected to one-way ANOVA to determine statistical significance. * *P* < 0.05, ** *P* < 0.01, *** *P* < 0.001

To investigate the major pathway by which RGS14 influences tumor behavior, we performed transcriptome sequencing of RGS14-knockdown (shRGS14) and shNC-transfected MHCC97H cells. KEGG enrichment pathway analysis revealed that RGS14 knockdown affects pathways involved in gap junctions, cell adhesion molecules, cAMP signaling, and calcium signaling (Fig. 5A). Reactome enrichment analysis revealed that RGS14 knockdown primarily influences GPCR ligand binding and extracellular matrix remodeling pathways (Fig. 5B). Given the central role of cAMP signaling in cellular communication, we evaluated the cAMP levels and activation of the downstream PKA‒CREB signaling pathway. Compared with control Huh7 cells, Huh7 cells overexpressing RGS14 presented elevated cAMP levels and increased phosphorylation of PKA and CREB proteins. The administration of the adenylate cyclase (AC) inhibitor SQ22536 to RGS14-overexpressing cells reversed the increase in cAMP and suppressed PKA/CREB phosphorylation (Fig. 5C, F; Supplementary Fig. 2D). Conversely, RGS14 knockdown in MHCC97H and SNU387 cells reduced the intracellular cAMP concentration and decreased p-PKA/p-CREB expression. Notably, treatment of RGS14-knockdown cells with the AC agonist forskolin restored cAMP levels and activated the downstream PKA‒CREB signaling pathway (Fig. 5D-E, G-H; Supplementary Fig. 2E, F). IHC analysis of RGS14 and p-CREB expression levels was performed on 88 HCC tissue microarrays (TMAs). The results revealed a significant positive correlation between RGS14 and p-CREB expression in HCC tissues (*R* = 0.254, *p* = 0.017; Fig. 5I, J), further confirming that RGS14 may influence HCC malignant progression through the PKA/CREB signaling pathway.

**Fig. 6:**
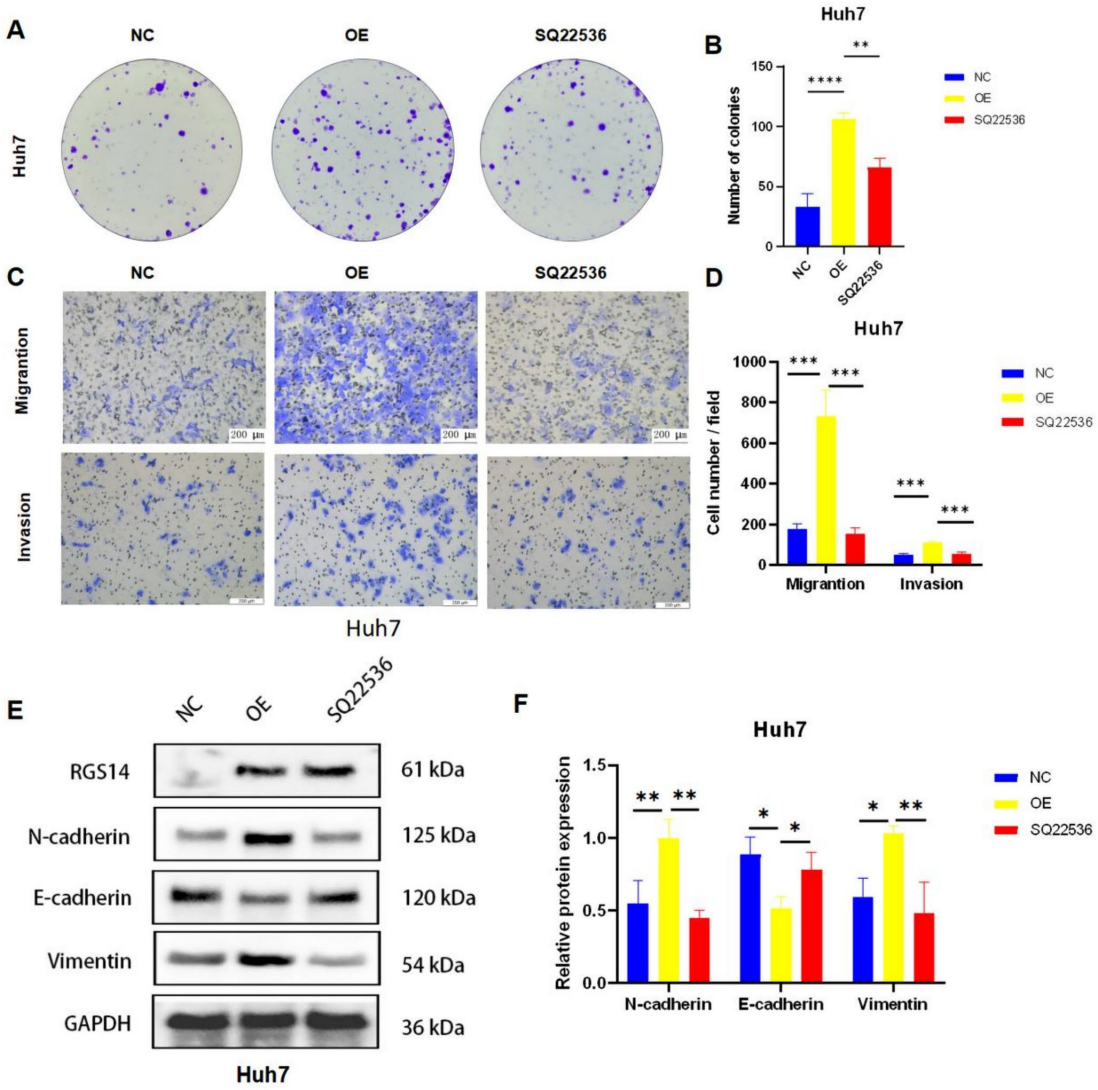
Rescue experiments employing cAMP inhibitors in RGS14-overexpressing Huh7 cells. (**A**, **B**) Colony formation assays were conducted to assess the proliferative capacity of RGS14-overexpressing Huh7 cells treated with the cAMP inhibitor SQ22536 (10µmol/L). (**C**, **D**) Transwell migration and invasion assays quantified the migratory and invasive potential of these cells following inhibitor treatment. (**E**, **F**) Western blot analysis revealed changes in EMT-related protein expression in RGS14-overexpressing Huh7 cell following treatment with the cAMP inhibitor SQ22536 (10µmol/L). This figure presents the mean values ± SD from three independent experiments. One-way ANOVA was employed to assess the statistical significance of the observed differences. * *P* < 0.05, ** *P* < 0.01, *** *P* < 0.001, **** *P* < 0.0001

To investigate the regulatory role of RGS14 in hepatocellular carcinoma progression through the cAMP/PKA/CREB axis, we used an AC inhibitor and agonist for experimental verification. Colony formation assays revealed that SQ22536 treatment abolished RGS14-mediated proliferation enhancement in Huh7 cells (Fig. 6A, B). Correspondingly, Transwell migration and invasion assays demonstrated that SQ22536 significantly suppressed RGS14-induced metastatic potential (Fig. 6C, D). Western blotting further revealed attenuation of RGS14-driven epithelial‒mesenchymal transition (EMT) in Huh7 cells following cAMP inhibition (Fig. 6E, F). Conversely, when the cAMP agonist forskolin was applied to RGS14-depleted MHCC97H and SNU387 cells, it restored their proliferative capacity, augmented their migratory and invasive capabilities, and reversed the suppression of the EMT process (Supplementary Fig. 3). These findings collectively establish that RGS14 modulates hepatic oncogenesis through cAMP level-dependent regulation of the PKA‒CREB signaling cascade.

### Knockdown of RGS14 inhibits tumor growth in vivo

**Fig. 7:**
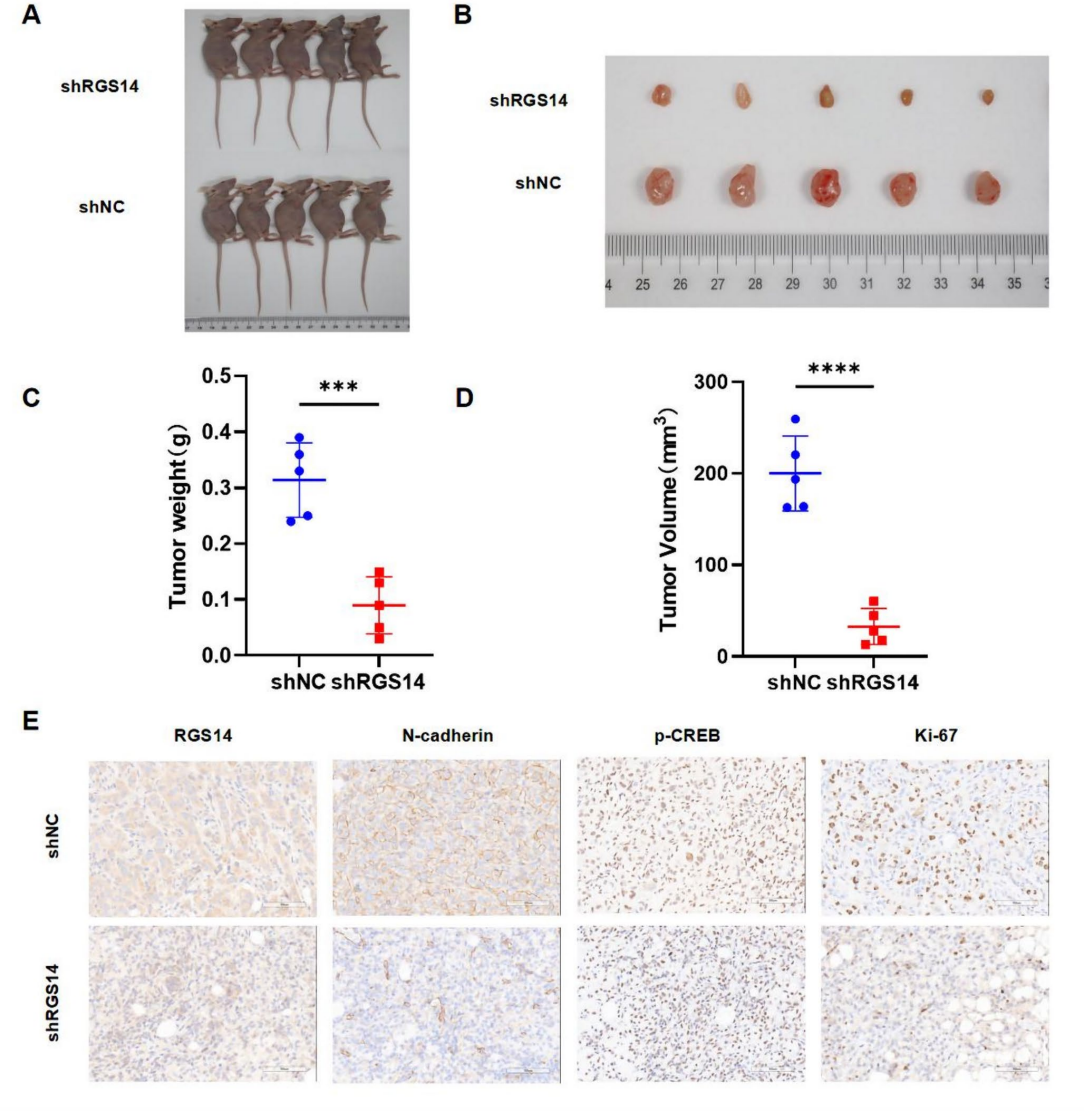
In Vivo Validation of RGS14’s Oncogenic Potential. (**A**) RGS14-knockdown or control MHCC97H cells were subcutaneously injected into nude mice (*N* = 5). (**B**) Representative images of xenograft tumors resected from nude mice 14 days post-inoculation. (**C**) Measurement of xenograft tumor weight (*N* = 5). (**D**) Tumor volume was measured at the end of the experiment (*N* = 5). (**E**) IHC analysis of RGS14, N-cadherin, p-CREB, and Ki67 protein expression in xenograft tumor tissues. Scale bar, 100 μm. *** *P* < 0.001, **** *P* < 0.0001

To further investigate the role of RGS14 in tumor progression, we utilized an in vivo xenograft model. BALB/c nude mice were subcutaneously injected with either RGS14-knockdown or control MHCC97H cells, and tumor growth was monitored over time. The results demonstrated that RGS14 knockdown significantly inhibited tumor growth (Fig. 7A, B). Compared with those in the control group, the tumor weight and volume in the RGS14-knockdown group were markedly lower (Fig. 7C, D). Immunohistochemical analysis revealed decreased expression levels of the mesenchymal marker N-cadherin, the proliferation marker Ki-67, and phosphorylated CREB (p-CREB) in the RGS14-knockdown group (Fig. 7E). These results suggest that RGS14 knockdown significantly inhibits tumor growth in vivo.

## Discussion

HCC is a highly malignant and heterogeneous tumor. Although surgical resection and liver transplantation play important roles in the treatment of HCC, postoperative recurrence and metastasis are key factors that negatively affect the long-term survival of patients. HCC is often diagnosed when the tumor is in later stages, and even macroscopic PVTT is found in 10–40% of patients (Lu et al. 2019). Therefore, studying the association between the formation of PVTT and the malignant progression of liver cancer, determining the potential mechanism that leads to HCC proliferation and metastasis, optimizing treatment plans and improving the prognosis of patients are urgently needed. Previous studies have shown that RGS proteins may play important roles in the malignant progression of HCC and the amelioration of immunotherapy responses (Hu et al. 2013; Ke et al. 2024; Wang et al. 2022; Zou et al. 2023). In this study, we found that RGS14 expression was significantly greater in HCC tissues than in adjacent liver tissues, especially PVTT tissues. In addition, increased RGS14 expression was significantly correlated with the TNM stage of HCC patients. Our data and TCGA data consistently revealed that HCC patients with high RGS14 expression exhibit significantly poorer clinical outcomes than those with low RGS14 expression. Importantly, RGS14 was an independent prognostic indicator of OS. Therefore, these results suggest that RGS14 can be used to predict the prognosis of HCC patients.

Due to their structural complexity and diversity, GPCRs form extensive and intricate signaling networks with G proteins, which may critically contribute to the modulation of tumorigenic properties. Recent studies have revealed the critical role of GPCRs in HCC tumorigenesis and progression (Peng et al. 2018). In contrast to the complex downstream signaling networks of GPCRs, RGS proteins exhibit highly tissue-specific expression patterns, suggesting that therapeutic targeting of these regulators during early-stage intracellular signal transduction may provide a more direct intervention strategy (Alqinyah and Hooks 2018). We performed a series of in vitro experiments to examine the effect of RGS14 on HCC malignant phenotypes. We found that RGS14 knockdown attenuated the proliferation, colony formation, migration and invasion of HCC cells and induced EMT in HCC cells. To investigate the role of RGS14 in HCC malignancy, we conducted a series of in vitro functional experiments. Our results revealed that increased RGS14 expression markedly amplified several key malignant traits in HCC cells, including enhanced proliferation, improved clonogenic survival, increased migratory and invasive abilities, and the activation of EMT. In contrast, RGS14 knockdown attenuated these phenotypic changes in HCC cells. These findings suggest that RGS14 can increase tumor proliferation, invasion and metastasis, thereby contributing to the maintenance of the malignant phenotype of HCC. Therefore, targeting RGS14 signaling may help to suppress the malignant phenotype of HCC.

cAMP is widely present in cells and regulates various biological processes or behaviors, including ion channel activation, gene expression, cell metabolism, cell differentiation and apoptosis (Chin et al. 2002). Previous studies have shown that RGS14 can bind to Gα family members by directly interacting with Giα1/3 (Brown et al. 2015), whereas Giα itself is known to suppress adenylate cyclase (AC) activity, thereby reducing the intracellular cAMP concentration (Baker et al. 1999). When PKA, which is the main cAMP-binding target protein, becomes activated, the regulatory subunit (R) dissociates from the catalytic subunit (C). The active catalytic subunit (containing the key Thr197 phosphorylation site) that is released phosphorylates a variety of substrates, such as CREB, PARP1, BAD and GSK3 (Zhang et al. 2024). Among these substrates, CREB can promote the proliferation, survival and migration of many types of cancer cells, and it is negatively correlated with the survival of cancer patients (Chowdhury et al. 2023). Interestingly, the PKA‒CREB signaling pathway, which is a major pathway that is regulated by cAMP, has been shown to promote the malignant progression of HCC (Hirsch et al. 2020; Kovach et al. 2006; Zhang et al. 2012). Several studies have demonstrated that increasing cAMP levels in HepG2 cells can suppress cell proliferation and induce cell cycle arrest. Specifically, this is characterized by a significant reduction in cyclin A expression; concurrent increases in p21, p27, and p53 expression; and altered intracellular localization of cyclin D1 (Lee et al. 1999; Massimi et al. 2017). However, treatment with vasoactive intestinal peptide (VIP) reduces cAMP concentrations, diminishes CREB phosphorylation at Ser133, and induces apoptosis in Huh7 cells via inhibition of the cAMP/CREB/Bcl-xL pathway; however, this effect can be abrogated by the addition of a cAMP antagonist (Hara et al. 2019). Given its diverse target sites, cAMP exerts multiple effects in HCC, suggesting that its promoting or inhibitory role may depend on the specific biological context. In this study, we found that RGS14 regulated cAMP levels, which in turn regulated PKA activity and downstream CREB phosphorylation and affected the EMT process in HCC cells. Moreover, the addition of a cAMP agonist or inhibitor to HCC cell cultures reversed the effects of RGS14 on HCC cell colony formation, invasion, migration and EMT, suggesting that RGS14 promotes HCC progression by activating this pathway.

Our findings establish that RGS14-mediated cAMP/PKA/CREB activation functionally contributes to HCC progression. In nonalcoholic fatty liver disease (NAFLD), the RGS14-Giα1/3 interaction enhances adenylate cyclase activity, increasing cAMP levels and stimulating AMPK signaling (Wang et al. 2024). However, its role in HCC remains unresolved—specifically, whether RGS14 recruits Giα1/3 via its RGS domain/GL motif to counteract Giα1/3-dependent cAMP suppression. Future research should prioritize deciphering these disease-context regulatory mechanisms.

## Conclusions and perspectives

In summary, high RGS14 expression can be used as a diagnostic and prognostic biomarker of HCC, and RGS14 may promote HCC progression by activating the cAMP/PKA/CREB signaling pathway. Our findings highlight the important role of RGS14 in regulating cAMP/PKA/CREB signaling activity in HCC cells, and these findings may contribute to the future development of therapeutic approaches that target RGS14.

## Electronic supplementary material

Below is the link to the electronic supplementary material.

Supplementary Material 1

Supplementary Material 2

Supplementary Material 3

Supplementary Material 4

Supplementary Material 5

## Data Availability

No datasets were generated or analysed during the current study.
