# Supplementary material for: RGS14 promotes the progression of hepatocellular carcinoma by activating the cAMP/PKA/CREB signaling pathway
Source: J Cancer Res Clin Oncol. 2025 May 2;151(5):153. doi: 10.1007/s00432-025-06212-y (PMC12045833; doi:10.1007/s00432-025-06212-y)
Supplement: Supplementary file 1 — Supplementary Material 1 [file 432_2025_6212_MOESM1_ESM.doc]

**Detailed information on shRNA**

**The sequences of shRNAs**

| NC shRNA | 5′- TTCTCCGAACGTGTCACGT-3′ |
| --- | --- |
| RGS14-shRNA1 | 5′-GGGCACAGCAGCTTCAGATCT-3′ |
| RGS14-shRNA2 | 5′- GCAGCAAATCTGAGAGCCACC-3′ |
| RGS14-shRNA3 | 5′- GGTCTACCTGGTGGGCAATGA-3′ |
