# Supplementary material for: RGS14 promotes the progression of hepatocellular carcinoma by activating the cAMP/PKA/CREB signaling pathway
Source: J Cancer Res Clin Oncol. 2025 May 2;151(5):153. doi: 10.1007/s00432-025-06212-y (PMC12045833; doi:10.1007/s00432-025-06212-y)
Supplement: Supplementary file 2 — Supplementary Material 2 [file 432_2025_6212_MOESM2_ESM.doc]

**Detailed information on antibodies and reagents**

| **Reagent** | **Company** | **Art.No.** | **Dilution concentration** |
| --- | --- | --- | --- |
| RGS14 | Proteintech Group | 16258-1-AP | 1 ：1500（WB） |
| E-cadherin | PTM BIO | PTM-5024 | 1 ：1000（WB） |
| N-cadherin | PTM BIO | PTM-5221 | 1 ：2000（WB） |
| Vimentin | PTM BIO | PTM-5376 | 1 ：2000（WB） |
| Occludin | Proteintech Group | 27260-1-AP | 1 ：5000（WB） |
| PKA C-α | CST | 4782S | 1 ：1000（WB） |
| p-PKA C(Thr197) | CST | 4781S | 1 ：1000（WB） |
| CREB | CST | 9198S | 1 ：1000（WB） |
| p-CREB(Ser133) | CST | 9197S | 1 ：1000（WB） |
| GAPDH | Proteintech Group | 10494-1-AP | 1 ：20000（WB） |
| Goat anti-Rabbit IgG antibody | PTM BIO | PTM-6261 | 1 ：10000（WB） |
| Goat anti-Mouse IgG antibody | Abbkine | A21010 | 1 ：25000（WB） |
| Forskolin | MCE | - | - |
| SQ22536 | MCE | - | - |
| RGS14 | Proteintech Group | 16258-1-AP | 1 ：200(IHC) |
| N-cadherin | PTM BIO | PTM-5221 | 1 ：1000(IHC) |
| p-CREB(Ser133) | CST | 9197S | 1 ：500(IHC) |
| Ki67 | Servicebio | GB111499-100 | 1 ：500(IHC) |
