# Supplementary material for: RGS14 promotes the progression of hepatocellular carcinoma by activating the cAMP/PKA/CREB signaling pathway
Source: J Cancer Res Clin Oncol. 2025 May 2;151(5):153. doi: 10.1007/s00432-025-06212-y (PMC12045833; doi:10.1007/s00432-025-06212-y)
Supplement: Supplementary file 4 — Supplementary Material 4 [file 432_2025_6212_MOESM4_ESM.pdf]

Editing Certificate

This document certifies that the manuscript

RGS14 promotes the progression of hepatocellular carcinoma by activating the cAMP/PKA/CREB signaling pathway

prepared by the authors

Xiangnan Liang

was edited for proper English language, grammar, punctuation, spelling, and overall style by one or more of the highly qualified English speaking editors at AJE.

This certificate was issued on **April 16, 2025** and may be verified on the AJE website using the verification code **085B-6E89-8392-EF0C-531P**.

Neither the research content nor the authors' intentions were altered in any way during the editing process. Documents receiving this certification should be English-ready for publication; however, the author has the ability to accept or reject our suggestions and changes. To verify the final AJE edited version, please visit our verification page at aje.com/certificate. If you have any questions or concerns about this edited document, please contact AJE at support@aje.com.

AJE provides a range of editing, translation, and manuscript services for researchers and publishers around the world. For more information about our company, services, and partner discounts, please visit aje.com.
